# SCN4A Channelopathies: From Disease Mechanisms to Variant Interpretation

**DOI:** 10.3390/genes17091116

**Published:** 2026-09-14

**Authors:** Paola D’Ambrosio, Lorenzo Cipriano, Alessia Pugliese, Roberta Petillo, Dario Ricciardi, Francesco Habetswallner, Carmelo Rodolico, Manuela Priolo

**Affiliations:** 1 Medical and Molecular Genetics, Azienda Ospedaliera di Rilievo Nazionale Cardarelli, 80131 Naples, Italy; lorenzo.cipriano@aocardarelli.it (L.C.);; 2 Department of Clinical and Experimental Medicine, University of Messina, 98125 Messina, Italy; 3 Clinical Neurophysiology Unit, Azienda Ospedaliera di Rilievo Nazionale Cardarelli, 80131 Naples, Italy

**Keywords:** SCN4A, NaV1.4, skeletal muscle channelopathy, variant interpretation, VUS, ACMG/AMP, gain of function, gating-pore current, loss of function, genotype-phenotype correlation

## Abstract

SCN4A encodes the skeletal-muscle voltage-gated sodium channel NaV1.4. Pathogenic variation in this gene produces fundamentally different disease mechanisms, including dominant alpha-pore gain of function, dominant S4 gating-pore currents, and reduced channel availability, with severe biallelic loss of function causing congenital myopathy and fetal hypokinesia. This heterogeneity makes variant interpretation difficult because formal variant classification, the direction of channel dysfunction, and patient-level disease attribution are closely related but distinguishable conclusions. In this review, we synthesize the clinical, genetic, electrophysiological, structural, and functional evidence relevant to SCN4A variant interpretation, with particular attention to missense variants of uncertain significance. We examine how inheritance, channel topology, phenotype, population data, segregation, RNA evidence, regional and residue-level context, and mechanism-matched functional assays can be integrated within the ACMG/AMP framework. Published pathogenic-enriched regions, gnomAD regional missense constraint, and same-residue observations may contribute to variant interpretation when the requirements of an applicable ACMG/AMP criterion are met; when they have not been specifically validated or calibrated for SCN4A, they are best used as contextual information to prioritize additional evidence generation. Based on this literature, we organize these evidence domains into a practical phenotype-first, mechanism-informed workflow for SCN4A variant interpretation. This workflow is not intended as an alternative to or extension of ACMG/AMP but as an SCN4A-specific application of established variant-interpretation principles in a gene associated with multiple inheritance patterns and directionally distinct disease mechanisms. After technical confirmation of a candidate variant, as well as phenotype and inheritance context guide selection of the relevant disease model, appropriately validated functional evidence may contribute directly to ACMG/AMP classification, while functional mechanism and patient-level disease attribution are documented as related but distinguishable interpretative outputs. The same approach applies when a variant is identified through genotype-first sequencing: interpretation should return to deliberate phenotyping, inheritance assessment, electrophysiological characterization, and consideration of the differential diagnosis before disease causality is inferred.

## 1. Introduction

Voltage-gated sodium channels convert membrane depolarization into the regenerative inward sodium current required for action-potential initiation and propagation. In adult skeletal muscle, NaV1.4, encoded by SCN4A (chr 17q23.3), is the predominant sodium-channel isoform. It is distributed throughout the sarcolemma and transverse-tubule system and is enriched at the neuromuscular junction, where it contributes to the safety margin that converts endplate depolarization into a propagating muscle action potential. NaV1.4 therefore links neuromuscular transmission to excitation-contraction coupling, and relatively small changes in channel gating or availability can profoundly alter muscle excitability [1,2].

The identification of an SCN4A pathogenic variant in hyperkalemic periodic paralysis in 1991 provided one of the earliest molecular demonstrations of a human ion-channel disorder [3]. SCN4A has since become a paradigmatic model of genotype–function–phenotype correlation. Disease-associated variants may cause either altered channel activation and fast/slow inactivation, perturb ion conduction, disturb proper protein membrane localization, or protein instability. Substitutions involving positively charged residues within S4 voltage-sensor segments may also generate an anomalous gating-pore current distinct from sodium conduction through the conventional alpha-pore [1,2,4].

This mechanistic diversity produces a broad and overlapping clinical spectrum. Dominant gain-of-function variants are typically associated with sodium-channel myotonia (SCM), paramyotonia congenita (PMC), and hyperkalemic or normokalemic periodic paralysis (HyperPP/NormoPP). Hypokalemic periodic paralysis type 2 (HypoPP2) most often results from neutralization of S4 gating charges and an aberrant gating-pore current. Rare loss-of-function variants can produce congenital myasthenic phenotypes, fetal hypokinesia, or congenital myopathy; the most severe congenital myopathy/fetal-hypokinesia spectrum is typically associated with biallelic loss of function, whereas the genetic architecture of reported myasthenic presentations is less uniform [1,2,4,5,6,7].

The increasing use of broad sequencing has shifted the principal challenge from variant detection to variant interpretation. Variant prioritization alone cannot establish the direction or clinical relevance of the functional effect on NaV1.4, which may involve alpha-pore gain of function, a gating-pore current, reduced channel availability, or no demonstrable disease-relevant functional abnormality. Existing reviews have established the biophysical and clinical foundations of SCN4A channelopathies in depth [1,2]. In this review, we therefore synthesize how phenotype, inheritance, channel mechanism, regional and residue-level context, segregation, and functional evidence can be integrated when evaluating an individual variant, rather than providing a comprehensive catalogue of SCN4A variants or disease-specific treatment recommendations. The workflow outlined here is not intended to replace, extend, or provide an alternative scoring system to ACMG/AMP. Rather, SCN4A is used as a disease-specific example to illustrate how established variant-interpretation principles can be applied when a single gene is associated with multiple inheritance patterns and directionally distinct molecular mechanisms. Variant classification, functional mechanism, and patient-level disease attribution are closely interrelated and may draw on overlapping evidence, but they remain distinguishable interpretative outputs that should be documented explicitly. The intended readership includes clinical geneticists, molecular diagnostic laboratories, neurologists, neuromuscular specialists, neurophysiologists, and research laboratories generating functional evidence. The workflow provides a shared structure for integrating evidence generated across these settings and does not imply that every evidence type must be generated by a single laboratory or clinical team.

## 2. Mechanistic Anatomy of NaV1.4

NaV1.4 is a pore-forming alpha-subunit composed of four homologous domains (DI–DIV), each containing six transmembrane segments (S1–S6). S1–S4 form the voltage-sensing module, whereas S5–S6 and the intervening P-loop form the central sodium-conducting pore. Positively charged residues in S4 move during depolarization and couple to pore opening through the S4–S5 linkers; the DI–DIII voltage sensors are primarily linked to activation, whereas DIV is more tightly coupled to fast inactivation. The intracellular DIII–DIV linker contains the conserved IFM motif that mediates rapid inactivation [1,2]. These elements are interpretatively important because pathogenic substitutions in different structural modules can perturb distinct channel processes rather than simply producing a generic ‘damaging’ effect.

**Figure 1:**
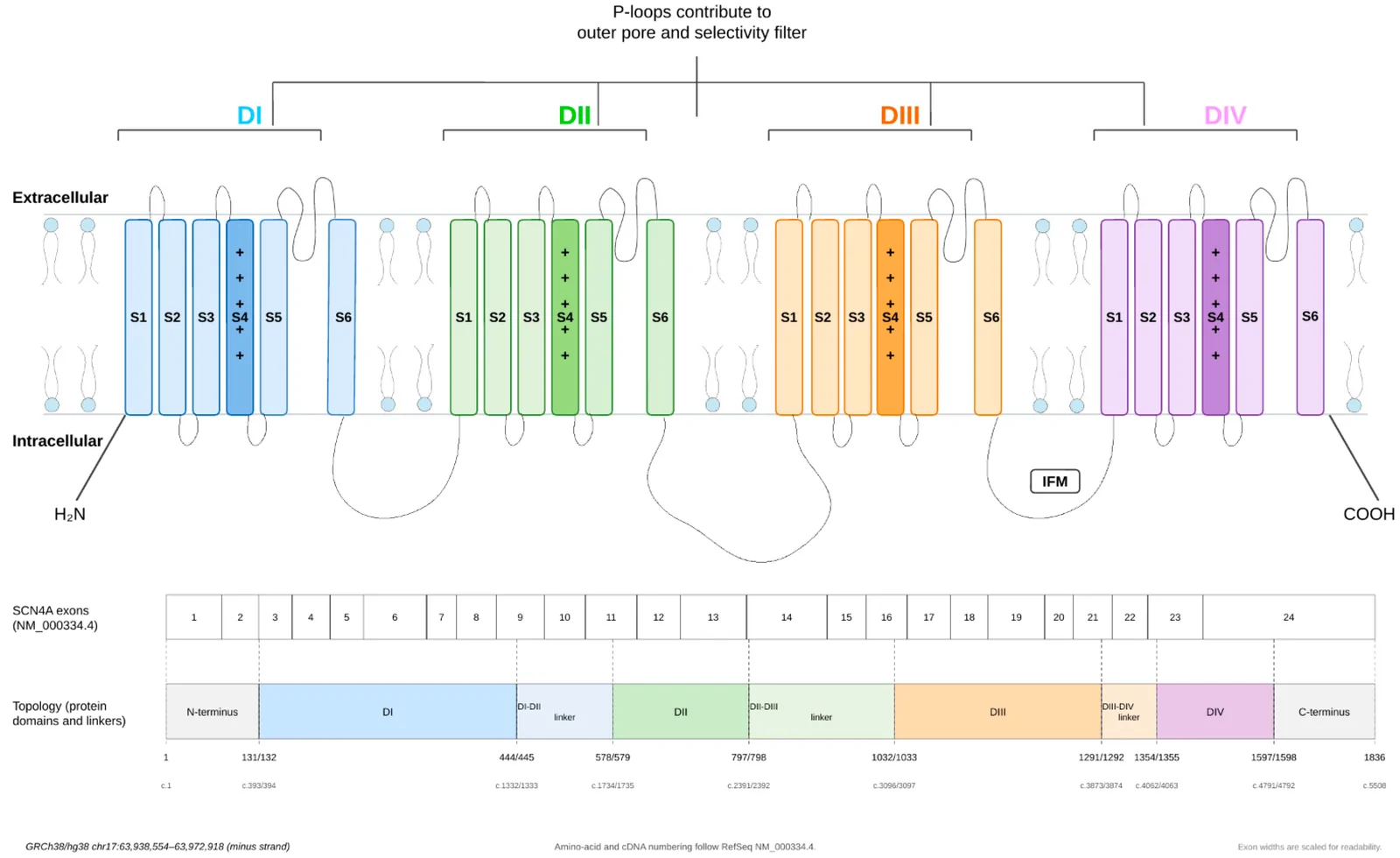
Structural organization of the skeletal-muscle voltage-gated sodium channel NaV1.4. NaV1.4 comprises four homologous domains (DI–DIV), each containing six transmembrane segments (S1–S6). S1–S4 form the voltage-sensing module, whereas S5–S6 and the intervening P-loop form the pore. Intracellular S4–S5 linkers couple voltage-sensor movement to pore opening, and the DIII–DIV linker contains the IFM motif involved in fast inactivation. The lower mapping panel aligns the 24 coding exons of SCN4A with NaV1.4 protein topology, indicating domain/linker boundaries in amino-acid and cDNA coordinates according to NM_000334.4; genomic coordinates refer to GRCh38/hg38.

NaV1.4 activates within milliseconds, generating the inward sodium current responsible for the rapid upstroke of the skeletal-muscle action potential. Fast inactivation limits current duration and contributes to membrane repolarization and refractoriness, whereas slow inactivation develops during prolonged depolarization or repetitive activity and regulates channel availability over longer time scales. Variants affecting S4 voltage sensors, pore-forming regions, S4–S5 linkers, or the DIII–DIV linker can disrupt this balance and provide the structural basis for the different SCN4A-related phenotypes [1,2,8]. These structural features and the exon-to-topology relationships used throughout this review are summarized in Figure 1.

## 3. Disease-Mechanism Classes Relevant to Variant Interpretation

Most dominant SCN4A channelopathies result from missense gain-of-function variants that increase inward sodium current through incomplete or delayed fast inactivation, persistent current or enhanced activation, accelerated recovery, or impaired slow inactivation. Modest excess inward current promotes repetitive firing and myotonia; more sustained depolarization can secondarily inactivate both mutant and wild-type channels, producing depolarization block and episodic weakness. Thus, the same primary gain-of-function direction can lead to either hyperexcitability or loss of excitability at the muscle-fiber level [1,2,4].

HypoPP2 is functionally distinct. Neutralization of positively charged S4 residues can create an anomalous ion-conducting pathway through the voltage-sensing domain rather than through the central alpha-pore. Under low extracellular potassium, this gating-pore current promotes paradoxical depolarization, resulting in NaV1.4 inactivation, and muscle-fiber inexcitability [1,2,4]. A standard alpha-pore assay may therefore fail to detect the disease-relevant defect [9].

Reduced channel availability forms a third interpretative class. It may result from impaired activation, enhanced inactivation, delayed recovery, defective membrane localization, splice-altering variants that reduce transcript output, or non-functional channels. Partial defects can reduce the safety margin for action-potential generation during repetitive activity and produce congenital myasthenic presentations. SCN4A-related congenital myasthenic syndrome has been reported with recessive loss-of-function alleles, but heterozygous pathogenic variants causing marked reduction in channel availability have also been described [2,6]. Severe biallelic loss of function is well established in fetal hypokinesia and congenital myopathy [1,2,7]. Accordingly, the inheritance model and allelic contribution in a myasthenic presentation should be established from segregation, phase, functional, and published evidence rather than inferred from the phenotype label alone.

## 4. Clinical Phenotypes as Mechanistic Readouts

The SCN4A clinical spectrum extends from myotonia to episodic or fixed weakness, neonatal respiratory crises, fatigability, and congenital myopathy. Diagnostic labels remain useful, but overlap is substantial; for variant interpretation, the phenotype is most informative when translated into the underlying excitability defect rather than treated as a mutually exclusive category [1,2,4,8].

SCM and PMC predominantly reflect muscle hyperexcitability. SCM causes delayed relaxation with variable warm-up and includes potassium-aggravated and severe myotonic subtypes, whereas PMC is characterized by paradoxical myotonia worsened by repeated activity and cold- or exercise-induced weakness. Severe neonatal gain-of-function presentations include neonatal myotonia and severe neonatal episodic laryngospasm (SNEL), in which stridor, choking, apnea, or life-threatening laryngeal crises may dominate early life [1,2,4,5,8].

Periodic paralysis reflects transient loss of muscle-fiber excitability. HyperPP/NormoPP usually causes relatively brief attacks triggered by potassium intake, fasting, cold, or rest after exercise and often coexists with myotonia. HypoPP2 typically causes longer attacks associated with low serum potassium, often after carbohydrate-rich meals or rest after strenuous exercise, and usually lacks interictal myotonia [1,2,4,8]. Congenital reduced-availability phenotypes may instead present with fatigable weakness, bulbar or respiratory involvement, fetal hypokinesia, hypotonia, contractures, or congenital myopathy depending on severity and allelic state [2,6,7]. The principal phenotype-mechanism, inheritance, electrophysiological, and histopathological relationships are summarized in Table 1.

## 5. Topology and Genotype–Function–Phenotype Correlations

Disease-associated SCN4A variants are distributed throughout NaV1.4 but are enriched in modules critical for voltage sensing, pore opening, and inactivation. Gain-of-function variants associated with myotonia frequently involve the DIII–DIV linker, intracellular S5–S6 regions, S4–S5 linkers, and the DIV voltage sensor. In a large Italian cohort, recurrent disease-associated regions included exons encoding DIIS4–S5, the DIII–DIV linker, DIVS4, and DIVS6; PMC-associated variants were relatively enriched toward the C-terminal portion of the channel, whereas SCM-associated variants were more broadly distributed [1,2,8].

HypoPP2 shows the clearest topographic-mechanistic relationship: established pathogenic variants neutralize outer S4 arginine residues in DI–DIII and generate a gating-pore current; no HypoPP2-causing S4 charge substitution has been established in DIV. HyperPP/NormoPP and PMC occupy a broader set of functional regions, including pore-forming segments, S4–S5 linkers, and DIVS4, where substitutions such as R1448 are strongly associated with altered fast inactivation [1,2,4,8].

**Figure 2:**
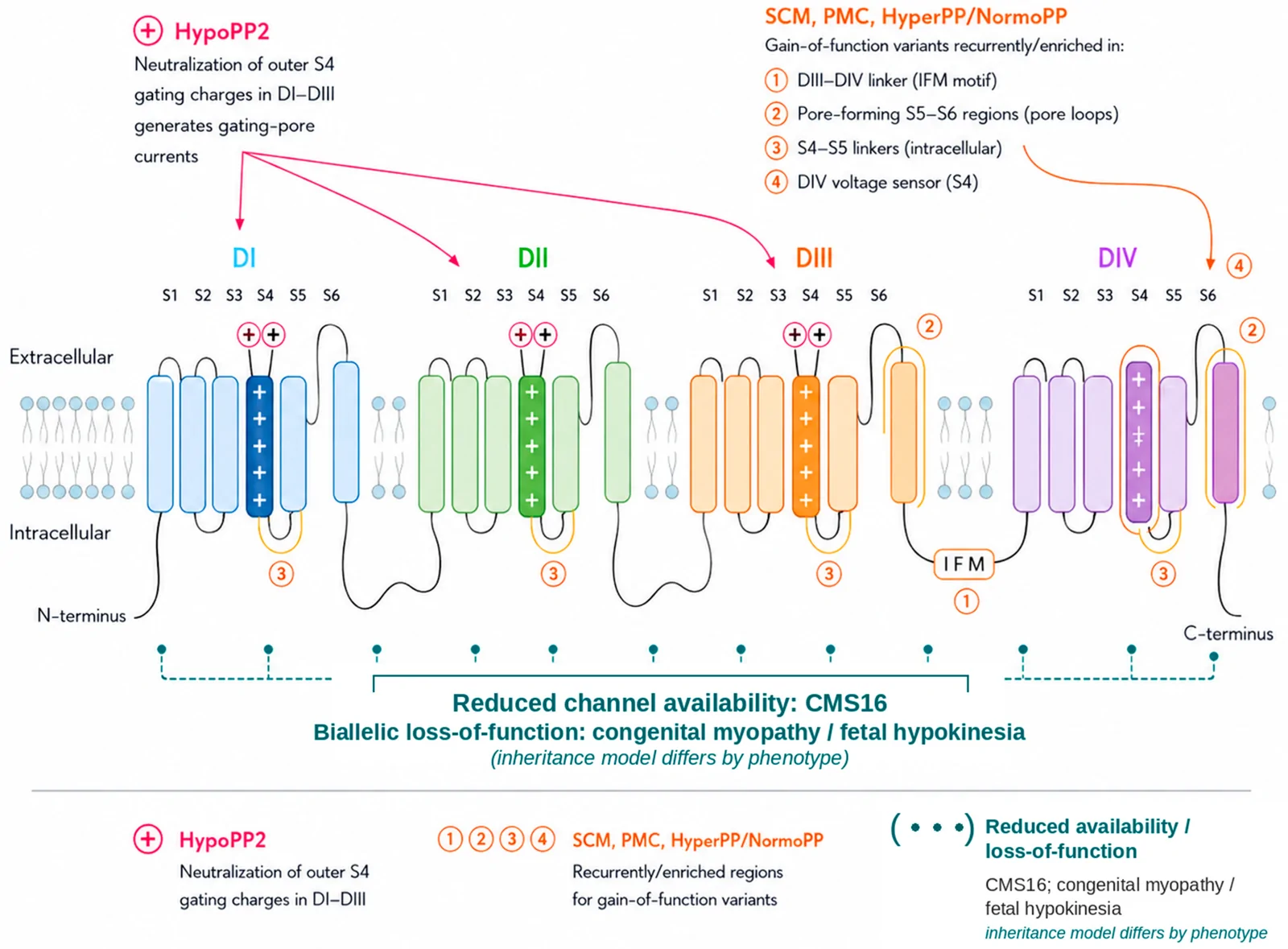
Domain-specific genotype–function–phenotype relationships in NaV1.4. Schematic representation of recurrent or enriched disease-associated regions. Gain-of-function variants associated with SCM, PMC, and HyperPP/NormoPP frequently affect the DIII–DIV linker, pore-forming S5–S6 regions, S4–S5 linkers, and DIV voltage sensor. Neutralization of outer S4 gating charges in DI–DIII generates gating-pore currents associated with HypoPP2. Reduced-availability variants associated with congenital myasthenic phenotypes and biallelic loss-of-function variants associated with congenital myopathy/fetal hypokinesia are indicated separately. Overlap across regions emphasizes that location informs, but does not independently determine, functional effect or phenotype.

Variant class and inheritance further constrain the plausible mechanism. Dominant myotonia and periodic paralysis are mostly caused by heterozygous missense variants that alter gating or generate a gating-pore current, whereas severe congenital myopathy and fetal hypokinesia are strongly associated with biallelic loss-of-function alleles. These correlations should not, however, be treated as deterministic for an individual variant: different substitutions at the same residue can produce distinct functional effects or phenotypes, and location alone cannot establish the molecular mechanism [1,2,6,7,8]. These domain-specific genotype–function–phenotype relationships are summarized schematically in Figure 2.

## 6. Variant Interpretation in SCN4A

### 6.1. Disease-Model-Specific Application of the ACMG/AMP Framework

As for other genes associated with multiple disease mechanisms, ACMG/AMP classification of SCN4A variants requires definition of the relevant gene-disease model, including the mode of inheritance, established molecular mechanism, and phenotype. SCN4A provides a particularly informative example because pathogenic variants in the same gene can act through dominant alpha-pore gain of function, dominant gating-pore currents, or reduced/lost channel function with distinct inheritance models and clinical consequences. Within the selected gene-disease model, the ACMG/AMP framework evaluates whether the available evidence supports classification of a variant as benign, likely benign, of uncertain significance, likely pathogenic, or pathogenic. Evidence informing the underlying molecular mechanism, including appropriately validated functional data, may contribute directly to this classification. The resulting variant class should nevertheless not be conflated with complete biophysical annotation of the channel defect or with the patient-level conclusion that the established variant-disease relationship explains the individual clinical presentation. Variant classification, functional mechanism, and patient-level disease attribution are therefore related but distinguishable outputs of an integrated interpretative process [10,11].

ClinGen has classified the relationship between SCN4A and autosomal recessive SCN4A-related myopathy as definitive, grouping congenital myasthenic syndrome, fetal hypokinesia, and congenital myopathy within a shared loss-of-function spectrum. This curation addresses the autosomal recessive disease model; dominant channelopathies and historical heterozygous myasthenic presentations therefore require a separate disease-model-specific interpretation. At the time of revision, no SCN4A-specific ACMG/AMP specifications developed by a ClinGen Variant Curation Expert Panel were listed in the ClinGen Criteria Specification Registry [12,13]. This does not mean that the ACMG/AMP framework is agnostic to inheritance: variant classification is performed within a defined gene-disease relationship, and the applicability or strength of individual criteria depends on the relevant mode of inheritance and disease mechanism. Allelic data, population-frequency expectations, segregation evidence, and predicted loss-of-function evidence may therefore have different relevance in dominant and recessive SCN4A models.

The mechanistic heterogeneity of SCN4A is particularly relevant to the disease-specific application of selected ACMG/AMP criteria. For PVS1, the relevant SCN4A-specific issue is selection of a disease model in which loss of function is established: predicted loss-of-function variants may contribute PVS1 evidence in recessive SCN4A-related myopathy, with evidence strength assigned according to transcript relevance, predicted nonsense-mediated decay, exon context, and expected residual function [14]. PVS1 is not applicable to dominant alpha-pore gain-of-function or gating-pore disease models. For PM1, generic location within a functionally important NaV1.4 region, such as an S4 segment, pore-forming region, S4–S5 linker, or DIII–DIV linker, is not by itself sufficient for criterion assignment; PM1 may nevertheless be applicable when a region has been adequately established as a mutational hotspot or critical functional domain with limited benign variation for the relevant disease model [15]. PP4 requires detailed phenotype and electrophysiological concordance with appropriate exclusion of competing diagnoses. A variant of uncertain significance (VUS) should not be treated as a partial molecular diagnosis or independently determine predictive testing, reproductive decisions, or disease-specific management; clinical management may nevertheless be warranted on the basis of the phenotype itself [2,8,10,16].

Population-frequency thresholds also require disease-model-specific calibration rather than a single gene-wide cutoff. For dominant gain-of-function or gating-pore phenotypes, population frequency should be judged against prevalence, penetrance, allelic heterogeneity, and the maximum plausible contribution of an individual variant to the relevant disease model. For recessive loss-of-function models, carrier frequencies that would be incompatible with a highly penetrant dominant disorder may remain compatible with pathogenicity when two disease-causing alleles are demonstrated in trans. Similarly, in silico missense predictors should be interpreted against calibrated, tool-specific thresholds for the applicable ACMG/AMP evidence strength rather than as binary deleterious/tolerant calls, and concordant outputs from correlated predictors should not be counted as independent evidence lines [16,17,18]. Disease-model-specific interpretative considerations are summarized in Table 2.

### 6.2. Functional, Computational, and Database Evidence

Functional evidence should be interpreted in relation to the mechanism interrogated by the assay. Functional studies are particularly informative in SCN4A when they address the disease-relevant channel mechanism. Gain-of-function phenotypes require assessment of activation; inactivation; persistent current; recovery; slow inactivation; use dependence; and, when relevant, temperature sensitivity. Suspected HypoPP2 variants require direct assessment of gating-pore current, whereas loss-of-function or reduced-availability phenotypes may require evaluation of current density, membrane expression, channel availability, recovery, use-dependent failure, or RNA splicing [1,2,4,6,7]. Their formal contribution to ACMG/AMP classification through PS3 or BS3 depends not only on mechanistic relevance but also on the degree of assay validation and calibration. A statistically significant difference from wild type alone is insufficient to justify PS3: the observed effect should be reproducible and biologically relevant, the assay should interrogate the appropriate disease mechanism, and the assigned evidence strength should reflect appropriate known pathogenic and benign controls, assay dynamic range, and demonstrated discriminatory performance. A normal or negative result in an assay that does not directly assess the disease-relevant mechanism should not be taken as evidence against pathogenicity for that mechanism; formal BS3 assignment additionally requires sufficient assay validation for the relevant class of dysfunction. Conversely, a mechanistically informative experiment may provide valuable biological insight even when the assay has not been sufficiently validated or calibrated to support formal PS3/BS3 evidence at a specified strength [19].

Computational predictions and public databases provide supporting rather than definitive evidence. General missense predictors cannot reliably distinguish gain of function, loss of function, or gating-pore behavior and should be used according to validated thresholds without counting correlated outputs independently [17]. Predicted splice effects should be assessed separately and, whenever possible, confirmed by RNA analysis [19]. ClinVar should be reviewed at the level of individual submissions, including condition, review status, date, evidence, and conflicts [20]. LOVD may identify previous observations, same-residue substitutions, phenotypes, and relevant publications, but entries should be traced to their primary source [21]. gnomAD is essential for evaluating ancestry-specific frequency, allele number, coverage, quality, and homozygous occurrence; absence from population databases supports rarity but does not establish pathogenicity [22]. Observations duplicated across ClinVar, LOVD, and the literature should not be counted as independent evidence.

### 6.3. Regional and Residue-Level Context in Variant Interpretation

Regional context has emerged as a complementary component of SCN4A missense-variant assessment. Pérez-Palma et al. defined pathogenic-enriched regions (PERs) through a gene-family burden analysis that aggregates missense variants across paralogous voltage-gated sodium-channel genes, including SCN4A, mapped onto a shared protein alignment. Within this framework, pathogenic/likely pathogenic missense variants localize preferentially to a limited number of structural domains, whereas benign and likely benign variants are largely excluded from these regions. Gene-specific analysis of SCN4A alone was not sufficient to identify PERs; these regions should therefore be interpreted as family-level annotations mapped onto NaV1.4 rather than as independently validated SCN4A-specific criteria [23]. Population-based constraint provides a complementary perspective. The gnomAD regional missense constraint (RMC) track identifies locally constrained segments in genes with regional variability in missense intolerance [22,24,25]. PERs and RMC are derived from different datasets and analytical approaches and should not be considered quantitatively equivalent. Regional annotations may contribute to ACMG/AMP evidence when the requirements of an applicable criterion are satisfied; in particular, PM1 depends on evidence that a defined region represents a relevant mutational hotspot or critical functional domain rather than on generic channel topology alone. In the absence of SCN4A-specific calibration, PERs and RMC provide useful regional context and can help prioritize additional segregation, RNA, or functional evidence.

Residue-level information is already incorporated into the ACMG/AMP framework through criteria applicable to previously established pathogenic missense substitutions affecting the same amino-acid residue; we therefore do not propose an additional residue-level pathogenicity criterion for SCN4A. When a VUS affects a residue at which a different missense substitution has already been established as pathogenic or likely pathogenic, this observation should be evaluated under the applicable ACMG/AMP criterion, including PM5 when its requirements are met [10,11]. The SCN4A-specific consideration is mechanistic: the precise functional consequence demonstrated for one amino-acid substitution should not automatically be assumed for every alternative substitution at the same position because different changes may alter NaV1.4 function to different degrees or through different functional consequences. Additional segregation or mechanism-matched functional studies may therefore remain useful for resolving uncertainty regarding the specific substitution or its mechanism.

In practice, regional and residue-level information should be assigned pathogenic weight when the requirements of the relevant ACMG/AMP evidence criterion are satisfied; otherwise, their principal value is to prioritize additional evidence generation. The present review does not derive SCN4A-specific likelihood ratios for PERs, RMC, or same-residue observations and should not be interpreted as demonstrating that these observations fail to meet quantitative ACMG/AMP evidence thresholds. In a highly suggestive phenotype, a rare missense variant within a constrained or pathogenic-enriched region, particularly at a residue already implicated by another disease-associated substitution, warrants higher priority for segregation, RNA, or mechanism-matched functional study. In a nonspecific phenotype, the same regional observations should not substitute for evidence supporting the relevant gene-disease model. Their practical value is therefore to identify which unresolved variants merit deeper investigation, while criterion-level pathogenic weight is assigned only when the applicable requirements are met.

Truncating and canonical splice-site variants require an interpretative approach distinct from that used for dominant missense gating variants. Severe reduction or loss of NaV1.4 function is particularly relevant to recessive congenital myopathy and fetal-hypokinesia phenotypes [1,2,7], whereas congenital myasthenic presentations require explicit establishment of the relevant allelic architecture and reduced-availability mechanism [2,6]. Truncating variants should therefore not be interpreted through the regional missense framework used for dominant gain-of-function channelopathies. Their assessment should instead consider zygosity and phase, transcript consequence, predicted nonsense-mediated decay, residual channel function, and the relevant disease model [14]. More broadly, these considerations emphasize that SCN4A variant interpretation is disease-model-specific, with missense gating variants and loss-of-function alleles requiring different combinations of evidence.

### 6.4. From Variant Classification to Patient-Level Interpretation and Family Counseling

Variant classification is the beginning rather than the end of the process of translating genomic information into clinical practice. Once a variant has been classified within a defined SCN4A gene-disease model, patient-level disease attribution should be considered separately. An atypical, mild, or absent phenotype in an individual carrier does not by itself alter an established variant classification; instead, patient-level discordance should prompt consideration of incomplete penetrance, variable expressivity, age-dependent manifestations, phenocopies, or alternative diagnoses. This assessment determines how well the established variant-disease relationship explains the proband and informs actionability, recurrence risk, testing of at-risk relatives, and phenotype-specific perioperative communication. Counseling is therefore a downstream consequence of the integrated interpretation rather than a separate variant-classification system.

For confirmed dominant SCN4A channelopathies, counseling should emphasize variable expressivity, 50% transmission probability, and phenotype-specific trigger and perioperative considerations. For established recessive loss-of-function disease, recurrence risk must be derived from parental genotypes. Phase determination and parental or partner testing are therefore integral to accurate counseling [26,27].

A VUS should be communicated as unresolved and should not independently justify predictive testing of unaffected relatives, disease-specific surveillance, reproductive decisions, or perioperative labeling, as widely acknowledged in international guidelines [10,28]. This does not mean that clinical precautions are withheld when the phenotype itself justifies them. In prenatal or neonatal settings, urgency does not lower the evidentiary threshold: an unresolved variant should not be converted into a prognostic result, whereas a confirmed diagnosis may appropriately inform delivery planning, respiratory support, or other phenotype-specific management [5,6,7,10,16]. The principal counseling implications are summarized in Table 3.

### 6.5. Practical Multidisciplinary Workflow for an SCN4A VUS

After technical confirmation of a candidate call, the interpretative logic should be phenotype-first and mechanism-aware: the clinical phenotype and inheritance context are used to select the relevant gene-disease model before criterion-level evidence is assigned. This does not imply a fixed chronological order of testing. A variant discovered through exome or genome sequencing still requires deliberate re-phenotyping and electrophysiological correlation before causality is inferred. Appropriately validated functional evidence may contribute directly to ACMG/AMP classification rather than representing a purely downstream step. The workflow below describes how evidence generated across clinical genetics, molecular diagnostic laboratories, neurology and neuromuscular practice, neurophysiology, and specialist research laboratories can be integrated; it does not require every team to generate every evidence type. Routine classification should use the evidence currently available, with unresolved variants remaining VUS. Family studies, RNA analysis, or mechanism-matched functional testing are escalation steps when feasible and when the result is expected to materially improve variant classification or patient-level interpretation. Formal ACMG/AMP classification, mechanism confidence, and patient-level disease attribution are related but distinguishable outputs of this integrated process [10,11,13,14,16,17,19,29].

*Box. Practical multidisciplinary approach to an SCN4A VUS*

1. Confirm the variant and allelic state (technical pre-interpretation step)

Confirm low-quality calls with an orthogonal method and report variants using HGVS nomenclature, the reference transcript, genomic coordinates, and zygosity. In suspected recessive SCN4A disease, establish phase and investigate variant classes not captured by the initial method when only one relevant allele is identified. This technical confirmation precedes but does not replace phenotype-first disease-model selection.

2. Define the clinical phenotype and inheritance context

Review age at onset, distribution of stiffness or weakness, warm-up or paradoxical myotonia, cold and exercise sensitivity, triggers, ictal potassium concentration, needle EMG, short- and long-exercise testing, repetitive nerve stimulation, respiratory involvement, congenital manifestations, and family history. Phenotype-first interpretation should guide both selection of the relevant SCN4A disease model and consideration of the broader differential diagnosis. Define the most plausible inheritance context and consider phenotype-appropriate alternatives, including CLCN1, CACNA1S, KCNJ2, and other genes relevant to the clinical presentation. The identification of an SCN4A VUS should not by itself establish disease attribution, particularly when phenotypic or electrophysiological concordance is incomplete or when alternative genetic explanations remain plausible. In such cases, other disease-associated genes and additional variant classes or alleles not captured by the initial testing strategy should be considered. This clinical context is used to select the relevant SCN4A disease model; it does not by itself establish variant pathogenicity.

3. Select the relevant SCN4A gene-disease/mechanism model

SCM, PMC, or HyperPP/NormoPP: predominantly autosomal dominant alpha-pore gain of function. HypoPP2: autosomal dominant gating-pore current, usually involving neutralization of an S4 gating charge. Congenital myopathy or fetal hypokinesia: typically biallelic loss of function. Reduced NaV1.4 availability should be considered in congenital myasthenic presentations; however, both recessive and heterozygous pathogenic variants have been reported, so the relevant inheritance model and contribution of individual alleles should be established from segregation, phase, functional, and published gene-disease evidence rather than assumed from the phenotype label [2,6]. Evaluate the variant against the specific gene-inheritance-mechanism-phenotype combination rather than against ‘SCN4A-related disease’ as a single entity.

4. Interrogate population, regional, database, and literature evidence within the selected disease model

Assess ancestry-specific frequency, allele number, coverage, quality metrics, and homozygous occurrence in gnomAD, including regional missense-constraint information when available. Determine whether a missense variant lies within a reported pathogenic-enriched or regionally constrained segment and whether other pathogenic or likely pathogenic substitutions have been reported at the same residue. Consider regional and same-residue observations under the applicable ACMG/AMP criteria when their requirements are met; otherwise use them as contextual information to prioritize additional evidence. Review condition-specific ClinVar submissions and trace relevant database observations to primary sources.

5. Evaluate segregation, de novo occurrence, and penetrance

Confirm biological relationships when using de novo evidence and establish the phase of variants in recessive disease. Clinically and electrophysiologically assess apparently unaffected carriers whenever possible. Interpret segregation and non-segregation in view of variable expressivity, incomplete or age-dependent penetrance, diagnostic uncertainty, and possible phenocopies. An apparently unaffected carrier should not be treated as automatic refutation of pathogenicity without considering expected penetrance and the strength of the underlying gene-disease model.

6. Match computational and functional evidence to the disease mechanism

Use calibrated missense and splice predictors without counting correlated outputs independently. When functional testing is required and available, select readouts capable of detecting the suspected alpha-pore gain of function, gating-pore current, reduced availability/loss of function, trafficking defect, temperature dependence, or splice alteration. Functional evidence should be interpreted in relation to the mechanism interrogated by the assay, while formal PS3/BS3 evidence strength should additionally reflect assay validation, appropriate pathogenic and benign controls, reproducibility, dynamic range, and discriminatory performance. A normal or negative result in an assay that does not directly assess the disease-relevant mechanism should not be taken as evidence against pathogenicity for that mechanism. Conversely, mechanistically informative data may remain biologically valuable even when assay calibration is insufficient for formal PS3/BS3 evidence at a specified strength.

7. Report the integrated interpretation using three explicit outputs

Document: (1) the formal ACMG/AMP variant classification within the specified disease model (benign, likely benign, VUS, likely pathogenic, or pathogenic); (2) mechanism confidence as supported, plausible, or unresolved; and (3) patient-level disease attribution/concordance as concordant, partially concordant, or discordant/unexplained. These latter categories are descriptive reporting terms rather than a validated scoring system. ‘Supported’ indicates direct variant-specific functional evidence or strongly convergent evidence establishing the disease-relevant defect; ‘plausible’ indicates a mechanism consistent with variant class, topology, phenotype, and/or partial functional evidence but not directly established for the specific variant; and ‘unresolved’ indicates that available evidence does not reliably distinguish among plausible mechanisms or is conflicting. Patient-level attribution is ‘concordant’ when phenotype, inheritance, and relevant electrophysiology fit the established variant-disease model without major unexplained discordance; ‘partially concordant’ when the core phenotype fits but one or more features are atypical, incomplete, or potentially influenced by variable expressivity or age; and ‘discordant/unexplained’ when the presentation is not adequately explained after considering penetrance, expressivity, and alternative diagnoses. Only the first output is a formal variant class. The latter two do not contribute additional ACMG/AMP points unless the underlying evidence independently satisfies an applicable criterion. A VUS classification means that the combined applicable ACMG/AMP evidence does not reach the threshold for likely pathogenic/pathogenic or likely benign/benign classification. Report uncertainty in mechanism and patient-level attribution separately and specify which additional evidence is most likely to resolve each uncertainty.

8. Translate the result into mechanism-specific counseling

Convey what the established disease model and patient-level interpretation imply for the individual and the family: recurrence risk, variable expressivity, the need for parental or partner testing, phase determination when relevant, and which relatives may benefit from targeted evaluation. For a confirmed diagnosis, document phenotype-specific perioperative considerations and trigger management for the anesthesia team without implying a universal anesthesia protocol. For an unresolved variant, state explicitly that the variant alone does not establish the diagnosis or justify predictive testing or disease-specific management and specify what additional evidence could resolve the classification or patient-level attribution.

## 7. Resolving Residual Uncertainty and Future Directions

After initial sequencing, additional testing should be selected according to the unresolved interpretative question rather than applied as a fixed sequence. Parental testing resolves phase, segregation, or de novo status. When the identified SCN4A variant does not adequately explain the phenotype, broader genomic evaluation may reveal an alternative diagnosis or an additional SCN4A variant not captured by the initial testing strategy, including a missing second allele, structural variant, or deep-intronic change. RNA studies address suspected splice or transcript effects; exercise testing or repetitive nerve stimulation can refine the physiological phenotype; and patch-clamp analysis can establish functional direction when the molecular mechanism remains uncertain [1,2,4,8]. Large SCN4A deletions or duplications have not emerged as a recurrent mechanism in the published skeletal-muscle channelopathy literature, although historical ascertainment limits how confidently rarity can be inferred.

RNA analysis is most useful when a specific transcript-level hypothesis exists. Because SCN4A is strongly enriched in skeletal muscle and is not detectably expressed in immune cells, peripheral-blood RNA is generally unsuitable for transcript-level assessment; muscle RNA or disease-relevant patient-derived cellular models are more informative [29,30,31,32].

Functional testing should define the precise consequence of individual variants when the molecular mechanism remains unresolved. Alpha-pore gain of function, gating-pore current, reduced channel availability, trafficking defects, and splice abnormalities require different approaches; the expression system, auxiliary subunits, ionic conditions, voltage protocol, and temperature should match the suspected mechanism. Functional data are most valuable when they resolve an uncertainty that clinical, familial, genomic, or RNA studies cannot. Mechanism-matched readouts identify the relevant biological question, whereas formal PS3/BS3 evidence strength additionally depends on assay validation and calibration as described in Section 6.2 [6,14,19,29]. Mechanism-relevant functional approaches are summarized in Table 4.

Future progress will depend on integrated genomic, transcriptomic, electrophysiological, structural, and biophysical evidence together with systematic sharing of standardized functional data. Automated electrophysiology and disease-relevant muscle models may increase scalability, but their value will depend on explicit assay validation for the mechanism being claimed. In this setting, precision medicine in SCN4A is currently best understood as increasingly precise mechanism-informed diagnosis and management rather than fully variant-specific therapy. Disease-specific treatment recommendations are beyond the scope of this review; nevertheless, established symptomatic approaches differ across channelopathy mechanisms and illustrate the downstream clinical relevance of defining functional direction [33,34,35,36]. Splice-directed approaches and patient-derived muscle models remain investigational tools for future mechanism-based research [37,38].

## 8. Conclusions

SCN4A channelopathies illustrate why disease-specific variant interpretation should be anchored to the relevant phenotype, inheritance pattern, and gene-disease mechanism. This principle is not unique to SCN4A; rather, SCN4A provides an instructive example because the same gene can produce dominant alpha-pore gain of function, dominant gating-pore currents, and reduced/lost channel function with distinct inheritance models and clinical consequences.

The practical implication is a phenotype-first, mechanism-informed application of the ACMG/AMP framework. Dominant missense variants should be evaluated within the relevant gain-of-function or gating-pore disease model, whereas truncating and other loss-of-function alleles require a loss-of-function model centered on zygosity, phase, transcript consequence, and residual function. Regional missense constraint, family-level pathogenic-enriched regions, topology, and same-residue observations may contribute to variant interpretation when applicable ACMG/AMP criteria are satisfied; when they have not been specifically validated or calibrated for SCN4A, they should primarily guide additional evidence generation rather than be assigned pathogenic weight automatically [22,23,24,25].

A robust integrated SCN4A interpretation should ultimately document three related but distinguishable outputs: the formal ACMG/AMP variant classification within the relevant disease model, the functional mechanism supported by the available evidence and the confidence in that assignment, and the degree to which the established variant-disease relationship explains the individual patient’s phenotype. These outputs arise from a single integrated interpretative process rather than from independent sequential assessments. This approach makes residual uncertainty explicit, targets additional testing, and supports counseling and diagnostic follow-up without creating an alternative scoring system. The SCN4A example may also be useful for other inherited channelopathies in which a single gene supports multiple, directionally distinct disease mechanisms.

## Figures and Tables

**Table 1:** Functional, inheritance, electrophysiological, histopathological, and selected clinical-management features of SCN4A-related phenotypes.

Functional Mechanism	Phenotype	Inheritance	Electrophysiology	Muscle Biopsy	Selected Clinical-Management Implications
Gain of function	Paramyotonia congenita (PMC)	AD	Myotonic discharges; paradoxical myotonia may be enhanced by repeated activity and cold; and the short exercise test typically shows a Fournier pattern type I, with CMAP reduction after exercise or cold.	Usually normal or nonspecific myopathic changes.	Cold avoidance, trigger recognition, and phenotype-specific clinical management.
Gain of function	Sodium-channel myotonia (SCM)	AD	Myotonic discharges; warm-up may occur. Exercise, cold, and potassium sensitivity vary by subtype; the short exercise test typically shows a Fournier pattern type III, with no CMAP reduction after exercise or cold.	Usually normal or nonspecific myopathic changes.	Document warm-up, pain, and trigger sensitivity; use phenotype-guided symptomatic management.
Severe gain of function	Neonatal myotonia/SNEL	AD, often de novo	Marked myotonic discharges may be present; laryngeal crises may dominate clinically.	Often normal or nonspecific.	Early recognition of laryngeal or respiratory crises and anticipatory respiratory planning.
Gain of function with secondary depolarization block	HyperPP/NormoPP	AD	Myotonia may be present interictally; reduced excitability or electrical silence may occur during attacks; and the long-exercise test may show an early CMAP amplitude increment and a late post-exercise decrement (Fournier pattern type IV).	Vacuoles and tubular aggregates may occur, particularly with chronic weakness.	Trigger and electrolyte counseling; monitor for fixed weakness over time.
S4 gating-pore current with secondary loss of excitability	HypoPP2	AD	Interictal myotonia is usually absent; the long-exercise test may show no early CMAP amplitude increment and a late post-exercise decrement (Fournier pattern type V); and muscle may become electrically inexcitable during attacks.	Vacuolar myopathy and tubular aggregates may occur.	Potassium- and trigger-related attack planning with phenotype-specific follow-up.
Loss of channel availability	SCN4A-related congenital myasthenic phenotype (CMS16)	Variable; recessive and heterozygous pathogenic variants have been reported	Decremental CMAP response to repetitive nerve stimulation; use-dependent failure may be prominent.	Variable and often nonspecific myopathic changes.	Assess bulbar and respiratory fatigability; individualized supportive management.
Biallelic loss of function	SCN4A-related congenital myopathy/fetal hypokinesia spectrum	AR	Myopathic EMG or reduced excitability; myotonia is generally absent.	Variable and often nonspecific congenital myopathic changes.	Multidisciplinary respiratory, feeding, and motor surveillance.

**Table 2:** Disease-model-specific considerations for SCN4A variant interpretation within the ACMG/AMP framework.

Disease-Mechanism Model	Typical Molecular/Structural Pattern	Mechanism-Matched Evidence	Main Interpretation Pitfall
Dominant alpha-pore gain of function (SCM, PMC, and HyperPP/NormoPP)	Usually heterozygous missense; recurrent regions include the DIII–DIV linker, S4–S5 linkers, intracellular S5–S6 regions, and DIV voltage sensor.	Activation/inactivation kinetics, persistent current, recovery, slow inactivation, use dependence, and temperature-sensitive protocols when relevant.	Generic topology alone is insufficient for PM1. Apply PM1 only when hotspot/critical-domain requirements are met; PS3/BS3 requires validated mechanism-relevant functional evidence [1,2,4,11].
Dominant S4 gating-pore current (HypoPP2)	Usually heterozygous missense neutralizing an outer S4 gating charge; DI–DIII outer S4 charges are particularly mechanism-informative.	Direct measurement of anomalous voltage-sensor leak current using protocols designed to isolate gating-pore conduction.	A conventional alpha-pore assay may not interrogate the disease-relevant gating-pore mechanism; a normal result therefore does not exclude pathogenicity through a gating-pore current [1,2,4].
Reduced channel availability/congenital myasthenic presentation	Variants causing reduced NaV1.4 availability; reported recessive and heterozygous cases mean inheritance and allelic contribution should be established from segregation, phase, functional, and published evidence.	Peak current density, availability, recovery, use-dependent failure, trafficking, and correlation with clinical electrophysiology.	Do not infer a uniform biallelic model from the phenotype label alone [2,6].
Biallelic loss of function (congenital myopathy/fetal hypokinesia)	Two pathogenic alleles in trans; missense, nonsense, frameshift, or splice-altering variants.	Current density, membrane expression, channel availability, RNA/splicing, and residual function.	Apply PVS1 only within an SCN4A disease model for which loss of function is established; it is not applicable to dominant alpha-pore gain-of-function or gating-pore mechanisms [7,18].

**Table 3:** Patient- and family-level counseling implications of an SCN4A variant result.

Disease Model	Recurrence Risk	Key Counseling Emphasis	Perioperative Communication	Family Testing
Dominant alpha-pore gain of function (SCM, PMC, and HyperPP/NormoPP)	50% per pregnancy	Variable expressivity dominates the conversation; inheritance predicts susceptibility to the mechanism, not a specific clinical course	Avoid depolarizing muscle relaxants where clinically appropriate; maintain normothermia and manage phenotype-specific triggers and electrolyte disturbances	Cascade testing may identify mildly affected or asymptomatic relatives who nonetheless benefit from perioperative documentation
Dominant S4 gating-pore current (HypoPP2)	50% per pregnancy	Attack triggers (carbohydrate load, rest after exertion) and potassium management are the actionable content; interictal myotonia is typically absent	Perioperative potassium monitoring; caution with glucose, insulin, and beta-adrenergic agents that lower serum potassium	Cascade testing informs relatives about trigger avoidance and perioperative precautions
Established recessive loss of function (congenital myopathy/fetal hypokinesia spectrum)	25% when both parents are heterozygous carriers; for an affected individual, offspring risk depends on partner carrier status	Parental testing and phase are required; outcome spectrum is broad and should not be presented as a single prognosis	Respiratory reserve and muscle excitability are the principal perioperative considerations; planning should be individualized to phenotype and functional status	Partner carrier testing may be relevant for an affected individual; heterozygous relatives carrying a clearly recessive allele are generally asymptomatic
Variant of uncertain significance (any model)	Not quantifiable from the variant alone	State explicitly that the variant is unresolved and should not independently determine management; phenotype-based precautions or management may still be appropriate.	No perioperative label follows from the unresolved variant alone; precautions should be based on the clinical phenotype when present	Targeted segregation studies may generate interpretative evidence, but predictive testing of unaffected relatives is not indicated

**Table 4:** Mechanism-relevant functional assays by suspected SCN4A disease mechanism. Formal PS3/BS3 evidence strength additionally depends on assay validation and calibration.

Suspected Mechanism	Primary Functional Readout	Expression System/Model	Interpretation Notes
Alpha-pore gain of function (SCM, PMC, and HyperPP/NormoPP)	Whole-cell patch clamp: shift of steady-state fast inactivation, slowed inactivation kinetics, and persistent or resurgent current	Heterologous expression (HEK293, CHO, and Xenopus oocytes) with co-expressed beta-1 subunit; temperature-controlled recordings for cold- or temperature-sensitive phenotypes	Match recording temperature and protocol to the clinical trigger (cold, exercise, and rest after exercise) whenever the phenotype is temperature- or activity-dependent
S4 gating-pore current (HypoPP2)	Detection of an anomalous ionic leak through the voltage-sensor domain at hyperpolarized or resting potentials, distinct from conduction through the central alpha-pore	Heterologous expression with ionic substitution protocols (e.g., guanidinium permeation) to isolate gating-pore from alpha-pore current	Requires protocols specifically designed to isolate gating-pore current; standard alpha-pore recordings alone may miss this mechanism
Loss of channel availability (CMS, fetal hypokinesia, and congenital myopathy)	Reduced peak current density, altered surface trafficking, or accelerated inactivation without a gain-of-function signature	Heterologous expression with surface-biotinylation or imaging-based trafficking assays; patient-derived muscle or myoblast cultures when available	Distinguish reduced current density caused by impaired trafficking from reduced availability caused by altered gating kinetics, because these represent different biological defects and may require different functional follow-up
Splice-altering or transcript-level variants	Aberrant transcript quantification and splice-junction usage	RNA obtained from muscle biopsy or disease-relevant patient-derived cellular models; blood RNA may be uninformative when SCN4A expression is insufficient for the question being tested	Most informative when the predicted splice effect cannot be resolved by computational prediction alone; interpret against a matched control transcript

## Data Availability

No new data were created or analyzed in this study. Data sharing is not applicable to this article.
